# In vivo imaging of renal microvasculature in a murine ischemia–reperfusion injury model using optical coherence tomography angiography

**DOI:** 10.1038/s41598-023-33295-9

**Published:** 2023-04-19

**Authors:** ByungKun Lee, Woojae Kang, Se-Hyun Oh, Seungwan Cho, Inho Shin, Eun-Joo Oh, You-Jin Kim, Ji-Sun Ahn, Ju-Min Yook, Soo-Jung Jung, Jeong-Hoon Lim, Yong-Lim Kim, Jang-Hee Cho, Wang-Yuhl Oh

**Affiliations:** 1 Department of Mechanical Engineering, KAIST, Daejeon, Republic of Korea; 2 KI for Health Science and Technology, KAIST, Daejeon, Republic of Korea; 3 Division of Nephrology, Department of Internal Medicine, Kyungpook National University Hospital, Daegu, Republic of Korea; 4 Cell and Matrix Research Institute, Kyungpook National University, Daegu, Republic of Korea

**Keywords:** Nephrology, Optics and photonics

## Abstract

Optical coherence tomography angiography (OCTA) provides three-dimensional structural and semiquantitative imaging of microvasculature in vivo. We developed an OCTA imaging protocol for a murine kidney ischemia–reperfusion injury (IRI) model to investigate the correlation between renal microvascular changes and ischemic damage. Mice were divided into mild and moderate IRI groups according to the duration of ischemia (10 and 35 mins, respectively). Each animal was imaged at baseline; during ischemia; and at 1, 15, 30, 45, and 60 mins after ischemia. Amplitude decorrelation OCTA images were constructed with 1.5-, 3.0-, and 5.8-ms interscan times, to calculate the semiquantitative flow index in the superficial (50–70 μm) and the deep (220–340 μm) capillaries of the renal cortex. The mild IRI group showed no significant flow index change in both the superfial and the deep layers. The moderate IRI group showed a significantly decreased flow index from 15 and 45 mins in the superficial and deep layers, respectively. Seven weeks after IRI induction, the moderate IRI group showed lower kidney function and higher collagen deposition than the mild IRI group. OCTA imaging of the murine IRI model revealed changes in superficial blood flow after ischemic injury. A more pronounced decrease in superficial blood flow than in deep blood flow was associated with sustained dysfunction after IRI. Further investigation on post-IRI renal microvascular response using OCTA may improve our understanding of the relationship between the degree of ischemic insult and kidney function.

## Introduction

Acute kidney injury (AKI) is a common risk factor associated with various etiologies of kidney dysfunction^1^. Despite continuing efforts to improve the diagnostic criteria for AKI^2,3^, it remains as a critical clinical problem without clearly defined therapeutic or preventive solutions. Various animal models for AKI have been developed to investigate the pathophysiology and identify new treatments. Among them, rodent ischemia–reperfusion injury (IRI) models have frequently been used to evaluate the basic mechanisms of AKI owing to their relatively reproducible nature^4–6^.

Ischemic injury triggers necrosis of proximal tubule cells, structural alteration of the endothelium, and inflammatory response^7,8^. Capillary rarefaction, a reduction in vascular density, is associated with renal fibrosis and progressive kidney dysfunction^9–11^. Decreased perfusion from capillary rarefaction can exacerbate tubular injury and induce chronic tissue hypoxia, which consecutively leads to irreversible fibrosis^12^. Microvascular changes after IRI have been studied using various diagnostic techniques, such as ultrasound, computed tomography, magnetic resonance imaging, and angiography^13–16^. Various indicators were introduced to evaluate microvascular distribution, such as quantitative analysis, perfusion signals, and fluorescence. However, changes in microvascular flow rate and the relationship between capillary blood flow and kidney injury are unknown in the IRI models.

Optical coherence tomography (OCT) is an imaging modality that can noninvasively visualize three-dimensional micron-scale structures of in vivo tissues. Although OCT has been most widely applied in ophthalmology and cardiology, limited number of studies have used OCT to image human kidneys^17,18^ and investigated the correlation between microtubular features and allograft function^19^. Advances in high-speed OCT in the last decade have enabled rapid repeated B-scanning of the same positions in the tissue, by which moving erythrocytes in the blood vessels can be separately highlighted. This emerging method, named OCT angiography (OCTA)^20,21^, provides label-free imaging of microvascular structures. Furthermore, since OCTA signal is positively correlated with the distance traveled by the erythrocytes, relative blood flow speeds can be distinguished if the time intervals between the repeated B-scans (interscan times) are adequately configured. When applied to a rodent kidney IRI model, this semiquantitative capillary flow speed imaging capability can provide useful information on the pathophysiology of ischemic AKI.

In this paper, we present a high-speed OCTA system for repeatable monitoring of the mouse kidney microcirculation and semiquantitative OCTA technique for microvascular flow speed estimation in a murine IRI model. Capillary flow speeds in the peritubular and glomerular vasculature were regularly evaluated during the first 60 mins after reperfusion in IRI with varying durations of ischemia using the OCTA system. The correlation between OCTA time-course measurements in the renal microvasculature upon reperfusion and later confirmed ischemic damage represented by biochemical and histopathological analyses was investigated.

## Materials and methods

### OCTA instrumentation

**Figure 1:**
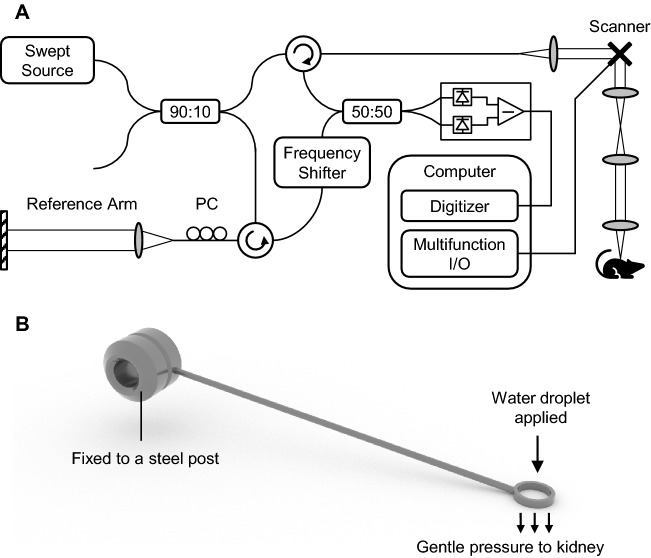
Optical instrumentation used for murine kidney OCTA imaging. (**A**) Schematic of the SS-OCT system operating at 1300-nm wavelength and 220.4-kHz A-scan rate. (**B**) Custom fixing tool designed for reducing pulsating and breathing motion of the kidney while imaging. Water droplet was applied inside the ring to move the reflective interface outside of the imaging window.

The raw data required for generating OCTA images were acquired using a previously reported swept-source OCT system^22^ based on a lab-built short-cavity laser with a 220.4-kHz sweep rate (Fig. 1A). The optical output of the laser was centered at 1300 nm with a 104-nm bandwidth, which corresponds to an 8.4-μm axial resolution in tissue. The scanning beam diameter was 4.9 mm at the 30-mm objective lens input, corresponding to an 8.2-μm transverse resolution in tissue. The optical interference signal was converted into an electric signal by a balanced receiver and recorded by a digitizer internally clocked at a 400-MHz sampling rate, resulting in a 3.3-mm digital imaging range. An acousto-optic frequency shifter was inserted in the reference arm to move the maximum coherence position away from the zero-delay line, thererby enhancing the signal at deeper imaging depths. The resulting system sensitivity was 102 dB, with a 6-dB roll-off measured at 3.2-mm imaging depth in the tissue.

### IRI induction and imaging protocol

A dorsal bilateral IRI model^23^ was established in 8-week-old male C57BL/6 mice. The mice underwent inhalation anesthesia (isoflurane 1–3%), followed by renal ischemia induction by clamping both renal arteries using a micro-serrefine with a 50–110 g pressing weight (RS-5422, Roboz Surgical Instrument Company, Inc. Gaithersburg, Maryland, USA). A similar surgical procedure without renal pedicle clamping was performed in sham-operated mice. During operation, the animals were maintained at 36.5–37 °C using a temperature-controlled heating device (Harvard Bioscience, Holliston, MD, USA). The mice were divided into the mild IRI group (*n* = 8) and moderate IRI group (*n* = 9) according to the duration of ischemia (10 and 35 mins, respectively). The kidneys were imaged using OCTA at baseline (before ischemia), during ischemia, and at 1, 15, 30, 45, and 60 mins after reperfusion. Because OCTA utilizes motion contrast, a ring-shaped custom fixing tool was developed to suppress breathing and pulsating motion (Fig. 1B). A water droplet was applied inside the ring to move surface specular reflections out of the OCT imaging depth window. All animal handling procedures were approved and supervised by the Korea Advanced Institute of Science and Technology Institutional Animal Care and Use Committee.

### Semiquantitative OCTA scanning and image processing

**Figure 2:**
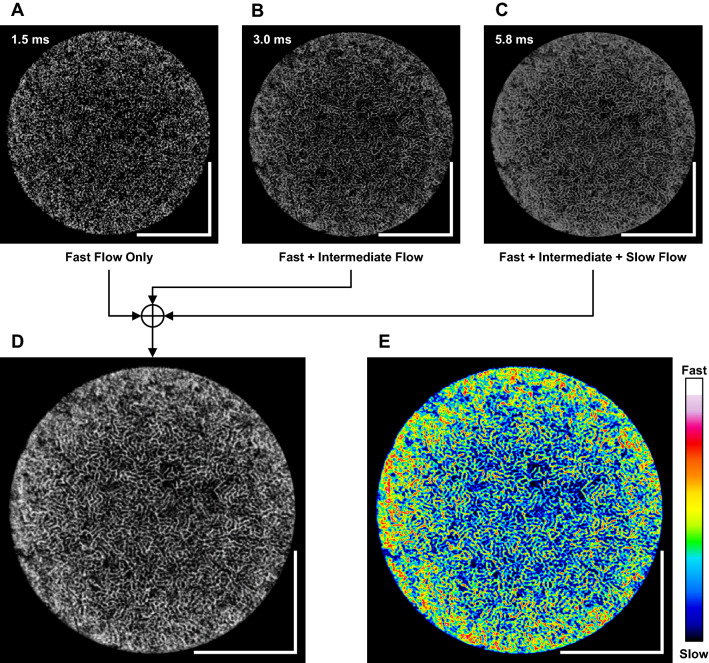
Semi-quantitative OCTA image processing. (**A**–**C**) Three raster scans with different inter-B-scan times were sequentially acquired over the same field of view. (**D**) *En face* projected three OCTA images were summated to generate semi-quantitative OCTA image. (**E**) False-color mapping helps visual perception of the fast and slow blood flow speeds. Scale bars 1 mm.

The OCTA scanning procedure and the image processing software were designed to achieve a roughly proportional relationship between the OCTA flow index and actual capillary flow speed^24,25^. Previous reports have shown that under the same imaging conditions, amplitude decorrelation—defined as the mean of normalized squared differences—shows a monotonic relationship with the ensemble mean of the distance traveled by the red blood cells up to a certain saturation distance determined by the optical resolution element^26,27^. Because the mean distance is proportional to the time interval between the two B-scans, the interscan time can be changed to adjust the measurable flow speed range. Therefore, we imaged the kidney using three raster scan patterns with different interscan times: raster scans comprising 256 × 256 A-scans (1.5-ms interscan time), 512 × 512 A-scans (3.0-ms interscan time), and 1024 × 1024 A-scans (5.8-ms interscan time), respectively (Fig. 2A–C). Each B-scan position was repeated three times, allowing two normalized squared difference images to be averaged to yield the OCTA signal. The maximum projection of the OCTA signals, located at superficial (50–70 μm from the surface) and deep (320–340 μm from the surface) depth ranges, measured from the automatically segmented kidney surface, were generated. For each depth range, the maximum OCTA projections obtained from the different scan patterns were summed to construct the final semiquantitative OCTA image (Fig. 2D). All three images (256 × 256, 512 × 512, and 1024 × 1024) were summed for the superficial capillaries, whereas only 256 × 256 and 512 × 512 images were summed for the deep capillaries because of OCTA signal saturation at high flow speeds. The semiquantitative OCTA images can be mapped onto pseudocolor to enhance the contrast for visual inspection (Fig. 2E). The flow index was defined as the total sum of OCTA signals across the circular region of interest. All flow indices were normalized with respect to the baseline value to remove the effect of intersubject variation in absolute blood flow.

### Renal function and histopathological studies

All mice were euthanized by cardiac puncture under anesthesia 7 weeks after reperfusion. Blood samples and the kidneys were collected for analysis. After centrifuging each blood sample, the serum underwent biochemical analysis. Blood urea nitrogen (BUN) and creatinine (Cr) levels were assessed by GC Labs (Yongin, Republic of Korea) using a Cobas 8000 modular analyzer system (Roche, Germany).

Kidney tissues from each experimental group were fixed with 4% paraformaldehyde (pH 7.4) and embedded in paraffin. 2-µm tissue sections were prepared and stained with Masson’s trichrome using standard protocols to determine histological changes and collagen deposition. Collagen deposition was quantified as a percentage of the total area using iSolution DT image software (IMT iSolution, Vancouver, Canada) in more than nine randomly selected fields in the cortex and medulla sections. For immunohistochemical staining, 2-μm-thick kidney sections were deparaffinized and rehydrated, and endogenous peroxidase was inactivated with 3% hydrogen peroxide. The samples were incubated with anti-CD31 (1:200; ab182981, Abcam) overnight at 4 °C and then detected using the EnVision-HRP kit (Dako, Carpinteria, CA, USA). To quantify CD31 expression, the marked cells were counted in nine non-overlapping fields imaged at 400 × magnification and divided by the area of the field of view for each slide.

### Statistical analysis

Data are specified as the mean ± standard error of the mean. Statistical analyses were performed using GraphPad Prism 5.01 (GraphPad Software, Inc., La Jolla, CA, USA). The difference in flow index between baseline and each reperfusion time was analyzed using the Wilcoxon test. The difference of the flow index between each datapoint and the baseline was analyzed using a nonparametric Mann-Whitney test with the significance level set at *p* < 0.05.

### Ethics approval

All animal handling procedures were approved by the Korea Advanced Institute of Science and Technology Institutional Animal Care and Use Committee and performed in accordance with the relevant guidelines and regulations. All procedures followed the recommendations of the ARRIVE guidelines^49^.

## Results

**Figure 3:**
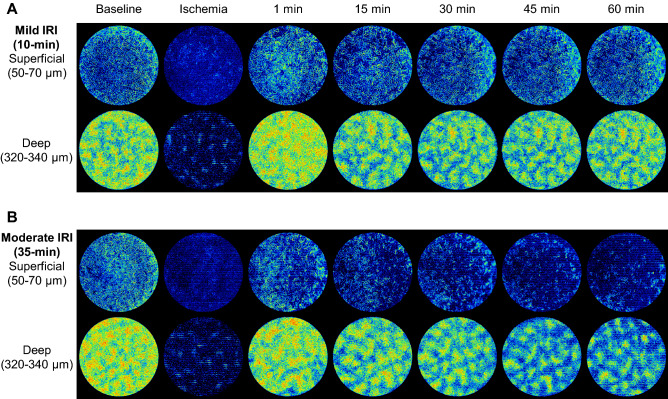
Results of the semi-quantitative OCTA blood flow measurement in the murine kidney IRI model. Representative time course of the OCTA signal in the superficial and the deep capillary layers of mild (10-min) and moderate (35-min) IRI groups.

Figure 3 shows representative examples of the semiquantitative OCTA blood flow measurements in the IRI model. The OCTA blood flow signal completely disappeared during ischemia in both the superficial and the deep layers. One minute after reperfusion, the blood flow recovered to a level similar to that at baseline in both layers.

**Figure 4:**
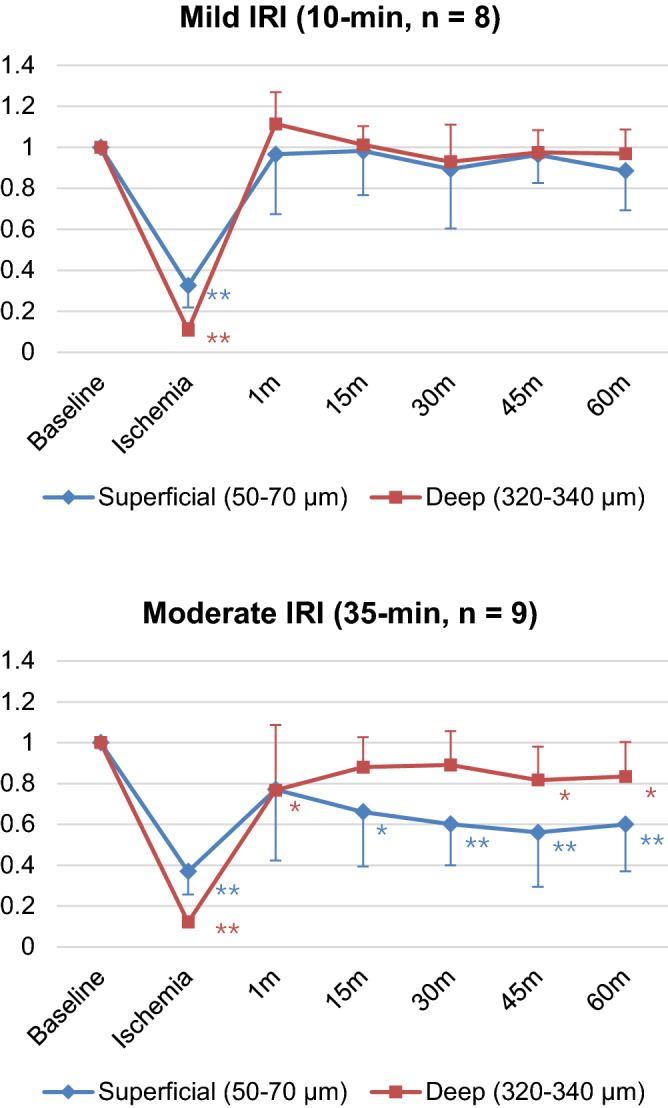
OCTA flow index with respect to the baseline in the mild and moderate IRI groups. *p < 0.05, **p < 0.01 vs. baseline. The flow indices were analyzed using a nonparametric Mann–Whitney test.

The behavior of the mean and its standard error of the flow indices in each group is shown in Fig. 4. OCTA flow index recovery showed contrasting trends between the mild and moderate IRI groups. The mild IRI group did not manifest a significant change in the OCTA flow index from baseline starting from 1 to 60 mins after reperfusion. The deep layer also did not show a decrease in flow at any time point after 1 min in the mild IRI group. In the moderate IRI group, superficial capillary flow recovered 1 min after reperfusion. However, superficial flow significantly decreased from 15 to 60 mins. Deep capillary flow presented a slightly delayed response, showing a significant decrease at 1 min and then at 45–60 mins after reperfusion. The amount of flow index change was more pronounced in the superficial layer than in the deep layer. Superficial blood flow was noticeably lower in the moderate IRI group than in the mild IRI group from 15 min after reperfusion. However, deep blood flow did not show a large difference between the mild and moderate IRI groups.

**Figure 5:**
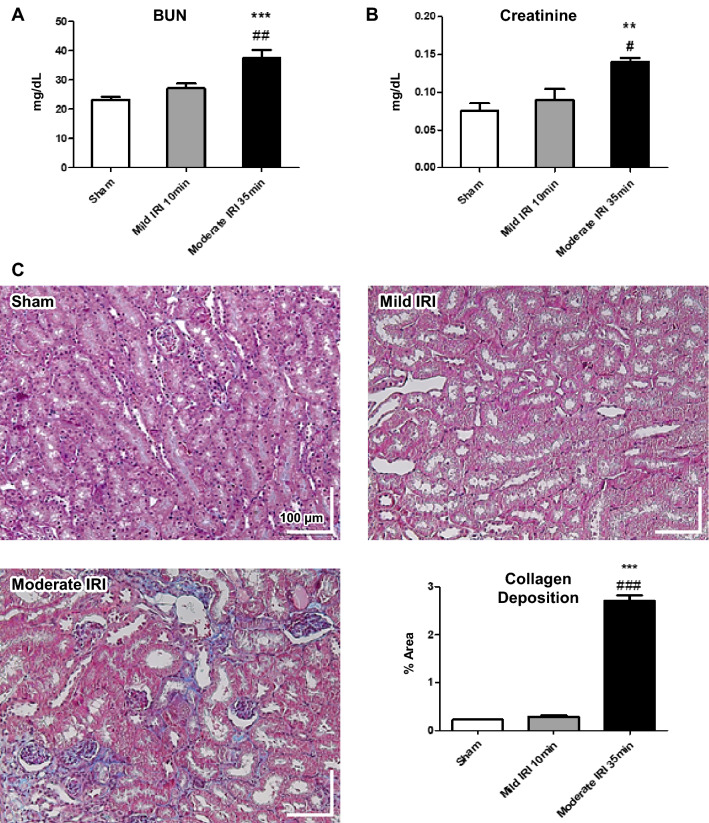
The kidney function and histological alteration in renal ischemia–reperfusion injury models 7 weeks after ischemia. (**A**, **B**) The levels of serum blood urea nitrogen (BUN) and creatinine were significantly increased in moderate IRI (35-min) group. (**C**) The trichrome-stained kidney sections (200×) and their quantifications showed tissue damage in renal tubular epithelial cells and increased collagen deposition in moderate IRI (35-min) group. Data represent mean ± SEM. *p < 0.05, **p < 0.01 ***p < 0.001 vs. sham; #*p* < 0.05, ##*p* < 0.01, ###*p* < 0.001 vs. mild IRI (10-min). The differences among the groups were analyzed using one-way nonparametric ANOVA followed by Tukey’s multiple comparison test.

**Figure 6:**
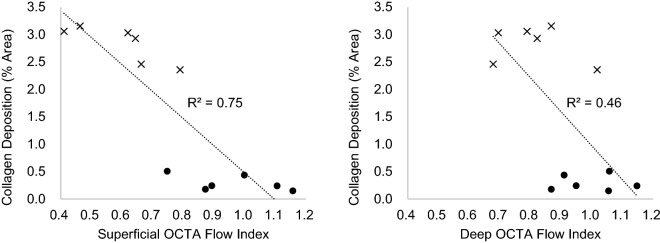
Correlation analysis between OCTA flow index and mean collagen area percentage in trichrome-stained slides. Collagen area was strongly correlated with superficial flow index and weakly correlated with deep flow index.

Seven weeks after IRI induction, biochemical blood tests and histological analyses were performed to confirm the relationship between ischemic injury and kidney function. The moderate IRI group showed a significant increase in serum BUN and Cr levels compared to the sham and mild IRI groups (Fig. 5A, B). Tubular damage and fibrosis were also increased in the moderate IRI group (Fig. 5C). Quantification of fibrosis by trichome staining showed significantly larger area of collagen deposition in the moderate IRI than in the mild IRI group. In order to investigate the relationship between reperfusion and kidney function, correlation analyses were performed between the superficial and deep OCTA flow indices, respectively, and the collagen area percentage (Fig. 6). The mean of the 45-min and 60-min flow indices were taken as a representative of the short-term capillary reperfusion. Superficial flow index exhibited a noticeably stronger correlation (R^2^ = 0.75) while deep flow index only showed a weak correlation (R^2^ = 0.46).

**Figure 7:**
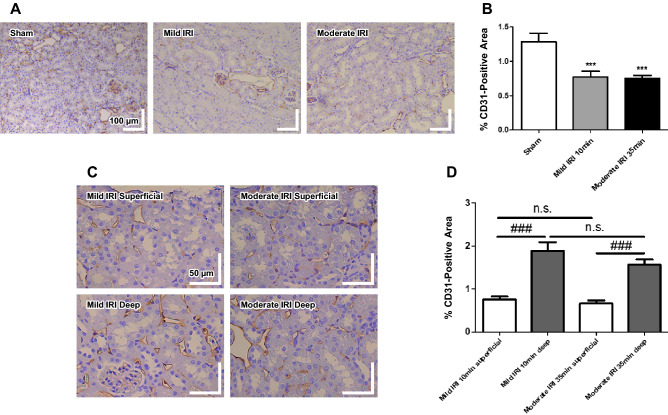
Immunohistochemical staining of CD31 in renal ischemia–reperfusion injury models 7 weeks after ischemia. (**A**, **B**) Immunohistochemical staining of CD31 was significantly decreased in the long-depth-range sections (200 ×) of both the mild IRI (10-min) and the moderate IRI (35-min) groups. (**C**, **D**) Quantitative analysis of smaller sections (400 ×) showed that CD31 was significantly more decreased in the superficial layer than in the deep layer. Data represent mean ± SEM. ***p < 0.001 vs. sham; ###*p* < 0.001 vs. deep layer; n. s. not significant. The differences among the groups were analyzed using a one-way nonparametric ANOVA followed by Tukey’s multiple comparison test.

Vascular damage was confirmed by quantification of CD31 immunostaining 7 weeks after ischemia. When a long depth range in the renal cortex was analyzed, vascular rarefaction was observed in both the mild and the moderate IRI groups (Fig. 7A). CD31 immunostaining decreased significantly in the mild and the moderate IRI groups compared to those in the sham group (Fig. 7B). When the renal cortex was divided into superficial and deep layers according to the OCTA depth range, the CD31-positive area was significantly smaller in the superficial layer than in the deep layer (Fig. 7C, D).

## Discussion

We established a high-speed OCTA system to measure the change in microvascular flow speed in a murine kidney IRI model. The OCTA system immediately detected the ischemia and the reperfusion status. Microvascular flow in the superficial and the deep layers showed different change patterns with respect to the duration of ischemia: superficial blood flow was more vulnerable to ischemic injury than deep blood flow. Changes in renal microvascular flow were associated with decreased kidney function, tubular damage, and collagen deposition 7 weeks after IRI. Therefore, this study suggests that OCTA can predict chronic kidney dysfunction through repeated noninvasive evaluation of ischemic injury.

Microvascular injury is a risk factor of progressive kidney dysfunction. IRI results in microvascular damage and reduces vascular density in the kidneys^28,29^. This capillary rarefaction is directly related to tissue hypoxia, which aggravates tubule necrosis and limits recovery from ischemic injury^30,31^. Microvascular damage has been mainly investigated in an animal model by either evaluating vascular resistance^32,33^ or calculating capillary density^34,35^. However, limitations of morphometric studies with kidney histology such as the invasive procedures and the difficulty to obtain repeated or functional data have complicated their application to human kidneys.

Various diagnostic methods have been studied to replace histological evaluation of microvascular injury after IRI^13–16^. However, these methods have disadvantages when applied in clinical practice. For example, computed tomography^14,36^ and ultrasound methods^13^ necessitate intravenous contrast agents. Contrast agents for computed tomography are nephrotoxic and dense microbubbles of ultrasound contrast decrease the intensity of deeper tissue. These make it difficult to perform repeated measurements in patients with AKI. Microangiography requires ex vivo preparation, making it an invasive and unrepeatable assessment^15,37^. Functional magnetic resonance imaging, a probable substitute for computed tomography, can evaluate perfusion or kidney volume but its resolution is insufficient for assessing microvascular flow distribution^16,38^.

OCT technology was applied to analyze the microscopic structure of the kidney in a noninvasive manner. OCT is a rapidly emerging noninvasive imaging technique which performs an “optical biopsy,” providing cross-sectional and three-dimentional tissue morphology without any contrast agents^39^. Wang et al., showed that OCT could provide real-time imaging of hypertrophic tubular structures in a rat model treated with intravenous Adriamycin^17,40^. They also performed a combined analysis of Doppler and structural OCT images to quantify blood flow in vivo^41^. Doppler OCT allowed noninvasive imaging of glomerular blood flow in rat kidneys. However, as discussed above, peritubular capillary rarefaction—rather than glomerular hypoxia—is a hallmark of the transition from AKI to chronic kidney dysfunction^42^. Deeper vascular beds are more difficult to assess with Doppler OCT because it requires relatively high signal-to-noise ratio and high field autocorrelation between adjacent A-scans. Also, Doppler OCT is not the optimal method for repeatable microvascular flow measurements due to its angle-dependence and insensitivity to transverse blood flow. Meanwhile, semi-quantitative OCTA can target microvascular distuributions of the interstitium, although some glomerular blood flow may be involved. Thus, compared to Doppler OCT, semi-quantitative OCTA can be considered a more suitable technology for measuring peritubular capillaries and estimating ischemic kidney injury.

Our OCTA flow index measurements showed greater ischemic damage to the glomerular capillaries in the superficial layer than to the peritubular capillaries in the deep layer. These results suggest that deep capillaries exhibit a higher level of recovery from ischemia. A possible explanation for this is that deep capillaries may receive more blood from the arcuate and the interlobular arteries of the medulla^43,44^. Moreover, immunohistochemical analysis via CD31 staining showed that the ischemic damage was negatively correlated with cortical depth. While previous studies had difficulties measuring the superficial and the deep cortices separately, our study has revealed that superficial capillaries are more susceptible to ischemic damage than deep capillaries using three-dimensional semi-quantitative OCTA.

The renal vasculature is characterized by a weak regenerative process, which causes persistent peritubular vascular rarefaction following significant ischemic injury^30^. Therefore, assessment of the vasculature at the time of kidney injury can predict long-term renal impairment. OCTA could be applied to kidney transplantion as allograft biopsy performed before and after kidney transplantation, potentially showing an association between peritubular capillary flow and perioperative AKI^45^. OCTA can safely estimate the peritubular flow of the allograft without an invasive procedure, such as tissue biopsy. Our study confirmed that the 60-min measurement of blood flow change was associated with kidney dysfunction and structural abnormalities, even after 7 weeks. To validate the results of this study, a controlled study of patients with AKI or those who have undergone kidney transplantation is needed to evaluate the predictive ability of OCTA for long-term kidney function.

Our semiquantitative OCTA technique is not free of limitations. One of the key limitations of OCTA-based flow speed measurements is its dependence on optical alignment. OCTA measures the blood flow speed based on optical decorrelation, which roughly represents the mean displacement of erythrocytes relative to the size of the resolution element. Because the illumination spot size increases with the axial distance from the focus, the OCTA flow index is less sensitive at depth positions farther from the objective focal plane. Therefore, to accurately measure fractional blood flow change across the IRI protocol timeline, the optical alignment of the animal with respect to the objective must be carefully controlled. Furthermore, the linearity of the correlative relationship between the OCTA flow index and the absolute blood flow velocity is yet to be thoroughly verified. Multiple flow phantom studies have suggested that a reasonably high degree of linearity can be achieved with accurate arithmetic calibrations^27,46–48^. However, these results must be interpreted with caution because flow index can also depend on the number density of the erythrocytes and the caliber of the vessel. These findings and the upcoming results from in vivo OCTA flow index studies can be adopted for future scan protocols and analysis software updates. Another limitation of our technique is the necessity of surgical procedures due to the limited OCT signal penetration into the tissue. Infrared light suffers from high scattering and absorption in common tissues such as stroma, requiring the imaging sample to be directly accessible through transparent media. OCTA imaging of the human kidney would thus be limited to intraoperative applications such as transplantation. Contrast-enhanced ultrasound is better suited for clinical studies of common kidney diseases since ultrasound echo signal can be detected from multiple centimeters of imaging depth.

In conclusion, this study demonstrated the efficacy of semiquantitative OCTA as a tool for assessing dynamic blood flow changes in murine kidney IRI models. OCTA flow index changes in superficial and deep capillaries showed contrasting results in the 10- and 35-min IRI models. Biochemical and histopathological analyses confirmed that a significant change in the OCTA flow index was indicative of decreased kidney function and vascular damage. These results suggest the potential of OCTA as a method for tracking relative blood flow changes in other mouse models of kidney injury and diseases. Improvements in the measurement protocol and flow index computation can lead to further validation of semiquantitative OCTA in kidney disease models.

## Data Availability

The datasets used and/or analyzed during the current study are available from the corresponding author on reasonable request.

## References

[1] Hoste EAJ (2018). Global epidemiology and outcomes of acute kidney injury. Nat. Rev. Nephrol..

[2] Kellum JA, Mehta RL, Angus DC, Palevsky P, Ronco C (2002). The first international consensus conference on continuous renal replacement therapy. Kidney Int..

[3] Mehta RL (2007). Acute kidney injury network: Report of an initiative to improve outcomes in acute kidney injury. Crit. Care.

[4] Zager RA, Johnson AC, Andress D, Becker K (2013). Progressive endothelin-1 gene activation initiates chronic/end-stage renal disease following experimental ischemic/reperfusion injury. Kidney Int..

[5] Wei Q, Dong Z (2012). Mouse model of ischemic acute kidney injury: Technical notes and tricks. Am. J. Physiol. Renal Physiol..

[6] Shiva N, Sharma N, Kulkarni YA, Mulay SR, Gaikwad AB (2020). Renal ischemia/reperfusion injury: An insight on in vitro and in vivo models. Life Sci..

[7] Ortiz A (2015). Translational value of animal models of kidney failure. Eur. J. Pharmacol..

[8] Pickering JW, Endre ZH (2014). The definition and detection of acute kidney injury. J. Renal Inj. Prev..

[9] Basile DP, Donohoe D, Roethe K, Osborn JL (2001). Renal ischemic injury results in permanent damage to peritubular capillaries and influences long-term function. Am. J. Physiol. Renal Physiol..

[10] Eardley KS (2008). The role of capillary density, macrophage infiltration and interstitial scarring in the pathogenesis of human chronic kidney disease. Kidney Int..

[11] Tanaka T, Nangaku M (2013). Angiogenesis and hypoxia in the kidney. Nat. Rev. Nephrol..

[12] Evans RG (2013). Haemodynamic influences on kidney oxygenation: Clinical implications of integrative physiology. Clin. Exp. Pharmacol. Physiol..

[13] Fischer K (2016). High-resolution renal perfusion mapping using contrast-enhanced ultrasonography in ischemia-reperfusion injury monitors changes in renal microperfusion. Kidney Int..

[14] Ehling J (2016). Quantitative micro-computed tomography imaging of vascular dysfunction in progressive kidney diseases. J. Am. Soc. Nephrol..

[15] Kramann R, Tanaka M, Humphreys BD (2014). Fluorescence microangiography for quantitative assessment of peritubular capillary changes after AKI in mice. J. Am. Soc. Nephrol..

[16] Tewes S (2017). Functional MRI for characterization of renal perfusion impairment and edema formation due to acute kidney injury in different mouse strains. PLoS ONE.

[17] Chen Y, Andrews PM, Aguirre AD, Schmitt JM, Fujimoto JG (2007). High-resolution three-dimensional optical coherence tomography imaging of kidney microanatomy ex vivo. J. Biomed. Opt..

[18] Andrews PM (2008). High-resolution optical coherence tomography imaging of the living kidney. Lab. Investig..

[19] Konkel B (2019). Fully automated analysis of OCT imaging of human kidneys for prediction of post-transplant function. Biomed. Opt. Express.

[20] Mariampillai A (2010). Optimized speckle variance OCT imaging of microvasculature. Opt. Lett..

[21] Kim DY (2011). In vivo volumetric imaging of human retinal circulation with phase-variance optical coherence tomography. Biomed. Opt. Express.

[22] Shin I, Oh WY (2020). Visualization of two-dimensional transverse blood flow direction using optical coherence tomography angiography. J. Biomed. Opt..

[23] Venkatachalam MA, Weinberg JM (2013). The conundrum of protection from AKI by adenosine in rodent clamp ischemia models. Kidney Int..

[24] Park T, Jang SJ, Han M, Ryu S, Oh WY (2018). Wide dynamic range high-speed three-dimensional quantitative OCT angiography with a hybrid-beam scan. Opt. Lett..

[25] Park JR, Lee B, Lee MJ, Kim K, Oh WY (2021). Visualization of three-dimensional microcirculation of rodents' retina and choroid for studies of critical illness using optical coherence tomography angiography. Sci. Rep..

[26] Richter D (2020). Relative retinal flow velocity detection using optical coherence tomography angiography imaging. Biomed. Opt. Express.

[27] Tokayer J, Jia Y, Dhalla AH, Huang D (2013). Blood flow velocity quantification using split-spectrum amplitude-decorrelation angiography with optical coherence tomography. Biomed. Opt. Express.

[28] Basile DP, Yoder MC (2014). Renal endothelial dysfunction in acute kidney ischemia reperfusion injury. Cardiovasc. Hematol. Disord.: Drug Targets.

[29] Babickova J (2017). Regardless of etiology, progressive renal disease causes ultrastructural and functional alterations of peritubular capillaries. Kidney Int..

[30] Basile DP (2011). Impaired endothelial proliferation and mesenchymal transition contribute to vascular rarefaction following acute kidney injury. Am. J. Physiol. Renal Physiol..

[31] Kwon O, Hong SM, Sutton TA, Temm CJ (2008). Preservation of peritubular capillary endothelial integrity and increasing pericytes may be critical to recovery from postischemic acute kidney injury. Am. J. Physiol.-Renal.

[32] Bonventre JV (1993). Mechanisms of ischemic acute renal failure. Kidney Int..

[33] Yagil Y, Miyamoto M, Jamison RL (1989). Inner medullary blood flow in postischemic acute renal failure in the rat. Am. J. Physiol..

[34] Kang DH (2001). Impaired angiogenesis in the aging kidney: Vascular endothelial growth factor and thrombospondin-1 in renal disease. Am. J. Kidney Dis..

[35] Kang DH (2001). Impaired angiogenesis in the remnant kidney model: I. Potential role of vascular endothelial growth factor and thrombospondin-1. J. Am. Soc. Nephrol..

[36] Braunagel M (2016). dynamic contrast-enhanced computed tomography: A new diagnostic tool to assess renal perfusion after ischemia-reperfusion injury in mice: Correlation of perfusion deficit to histopathologic damage. Invest. Radiol..

[37] Advani A (2011). Fluorescent microangiography is a novel and widely applicable technique for delineating the renal microvasculature. PLoS ONE.

[38] Hueper K (2014). Acute kidney injury: Arterial spin labeling to monitor renal perfusion impairment in mice-comparison with histopathologic results and renal function. Radiology.

[39] Fujimoto JG (2003). Optical coherence tomography for ultrahigh resolution in vivo imaging. Nat. Biotechnol..

[40] Wang B (2017). Optical coherence tomography and computer-aided diagnosis of a murine model of chronic kidney disease. J. Biomed. Opt..

[41] Wierwille J (2011). In vivo, label-free, three-dimensional quantitative imaging of kidney microcirculation using Doppler optical coherence tomography. Lab. Investig..

[42] de Braganca AC, Volpini RA, Mehrotra P, Andrade L, Basile DP (2016). Vitamin D deficiency contributes to vascular damage in sustained ischemic acute kidney injury. Physiol. Rep..

[43] Martinoli C, Bertolotto M, Pretolesi F, Crespi G, Derchi LE (1999). Kidney: Normal anatomy. Eur. Radiol..

[44] Yoldas A, Dayan MO (2014). Morphological characteristics of renal artery and kidney in rats. Sci. World J..

[45] Doreille A (2021). Acute kidney injury, microvascular rarefaction, and estimated glomerular filtration rate in kidney transplant recipients. Clin. J. Am. Soc. Nephrol..

[46] Su JP (2016). Calibration of optical coherence tomography angiography with a microfluidic chip. J. Biomed. Opt..

[47] Lee HJ, Samiudin NM, Lee TG, Doh I, Lee SW (2019). Retina phantom for the evaluation of optical coherence tomography angiography based on microfluidic channels. Biomed. Opt. Express.

[48] Choi WJ (2016). Characterizing relationship between optical microangiography signals and capillary flow using microfluidic channels. Biomed. Opt. Express.

[49] Percie-du-Sert N (2020). Reporting animal research: Explanation and elaboration for the ARRIVE guidelines 2.0. PLoS Biol..

